# An Interesting Case of Multiple Epiphyseal Dysplasia Masquerading as Myopathy in a Southeast Asian Girl

**DOI:** 10.7759/cureus.78400

**Published:** 2025-02-02

**Authors:** Shikha Swaroop, Preeti Srivastava, Kumar Diwakar, Jayanta K Laik, Minakshi Mishra, Rishi Anand

**Affiliations:** 1 Department of Pediatrics, Manipal Tata Medical College, Manipal Academy of Higher Education (MAHE), Jamshedpur, IND; 2 Department of Pediatrics, Tata Main Hospital, Jamshedpur, IND; 3 Department of Joint Replacement and Orthopedics, Tata Main Hospital, Jamshedpur, IND; 4 Department of Pathology, Tata Main Hospital, Jamshedpur, IND; 5 Department of Anesthesiology, Manipal Tata Medical College, Manipal Academy of Higher Education (MAHE), Jamshedpur, IND; 6 Department of Anesthesiology, Tata Main Hospital, Jamshedpur, IND

**Keywords:** autosomal dominant, multiple epiphyseal dysplasia, myopathy, skeletal dysplasia, waddling gait

## Abstract

Multiple epiphyseal dysplasia (MED) though one of the common skeletal dysplasias leads to a diagnostic dilemma. This is because the initial presentation is subtle in most of the cases which mimics other disorders like myopathies and rheumatological conditions. We present a case of a ten-year-old girl whose initial symptoms led to an extensive workup of myopathies yielding no conclusion. The final answer to the diagnosis was found through a genetic test. Most of the MED cases are because of mutation in the autosomal dominant gene which has variable penetrance leading to the absence of any family history. In this case too, family history was not significant.

## Introduction

Skeletal dysplasias are rare, chronically debilitating diseases affecting bone growth, with no current treatments. Multiple epiphyseal dysplasia (MED) is a common skeletal dysplasia affecting approximately 1 in 10000 individuals [1]. Initial symptoms are subtle often leading to misdiagnosis. The onset of the disease is in childhood with symptoms like waddling gait and difficulty in climbing stairs [2]. Long bones of our body grow by endochondral ossification. In MED, this ossification is defective [3]. MED results from mutations in genes encoding cartilage extracellular matrix proteins, enzymes, and transporter proteins. Defective cartilage protein makes epiphyses susceptible to wear and tear. Defective cartilage mineralization at long bone ends causes joint pain, deformity, and early osteoarthritis [4]. Mutations in several genes cause this disease [5]. We report a case of MED with an initial presentation mimicking myopathy. Genetic testing established the final diagnosis.

## Case presentation

A ten-year-old girl presented with a history of early fatigue during cycling for one year compared to siblings, frequent falls while walking, and difficulty rising from a seated position. Birth and developmental histories were normal. Her parents were healthy. She had one younger sister and one younger brother, both healthy with no similar complaints. The child was asymptomatic until eight years of age. On examination, the girl was alert and cooperative. Her height (146 cm) and weight (40 kg) were one standard deviation above the mean. The upper segment to lower segment ratio was normal. The central nervous system examination revealed right upper thigh tenderness and 4/5 power with normal tone in the right knee flexor muscle group. Power and tone in other muscle groups were normal. Deep tendon reflexes at all joints were normal. She exhibited a waddling gait and knee valgus deformity. All other joint examinations were normal, with no contractures. Other neurological examinations were normal. Orthopedic opinion was taken and no intervention was suggested from their side. Based on the initial presentation, differential diagnoses included inflammatory myopathy (myositis), congenital myopathy/dystrophy, and mitochondrial myopathy. Lab investigations and imaging studies are detailed in Table 1.

**Table 1:** Laboratory results in a patient with suspected myopathy and multiple epiphyseal dysplasia NCV: Nerve Conduction Velocity Study; RNST: Repetitive Nerve Stimulation Study

Lab investigation	Values	Normal range
C-reactive protein	0.87	0.02-1.0 mg/dl
Creatinine phosphokinase	123	45-95 U/l
Vitamin D	23	10-50 ng/l
Serum calcium	9.6	8.8-10.8 mg/dl
Vitamin B12	203	180-914 pg/l
Thyroid-stimulating hormone	2.8	0.3-6 mIU/l
Anti-Jo antibody	Negative	
Anti-nuclear antibody	Negative	
HLA B27	Negative	
ECG	Normal	
ECHO	Normal	
MRI of both thighs	Mild decrease in the bulk of the right thigh	
NCV	Bilateral lower limb normal	
RNST	Normal	

The laboratory and imaging investigations provided several key findings. The C-reactive protein level was measured at 0.87 mg/dl, indicating no significant inflammation. The creatine phosphokinase level was slightly elevated at 123 U/l; however, this mild elevation is not significant enough to confirm or rule out myopathy. Vitamin D levels were found to be 23 ng/ml, and serum calcium was 9.6 mg/dl, both of which were within normal limits. The vitamin B12 level was 203 pg/ml, again indicating no abnormalities. The thyroid-stimulating hormone level was recorded at 2.8 mIU/l, suggesting normal thyroid function.

**Figure 1:**
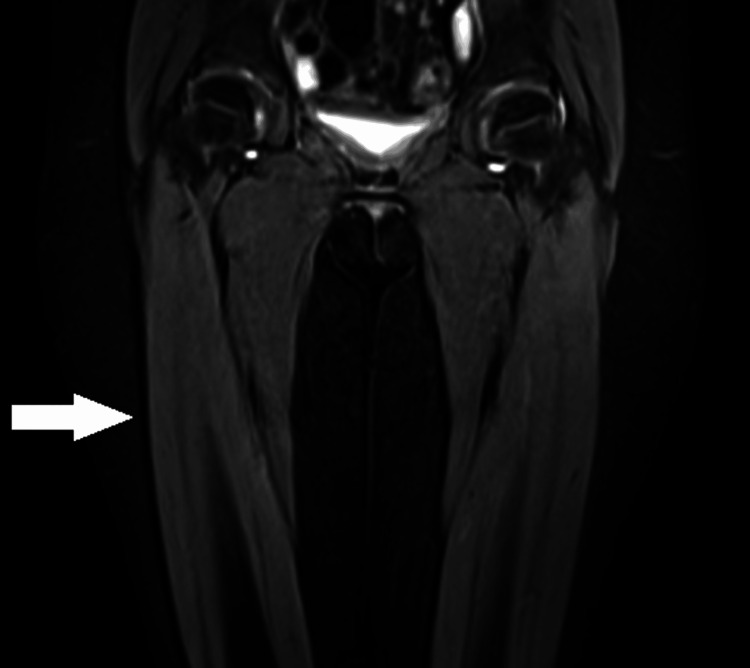
MRI of both lower limbs showing decreased muscle bulk in the right thigh

Autoantibody tests returned negative results for anti-Jo antibody, anti-nuclear antibody, and HLA B27, which suggests no significant autoimmune activity. Both the ECG and ECHO were reported as normal, indicating no immediate cardiac concerns. An MRI of both thighs showed a mild decrease in the bulk of the right thigh, which may require further evaluation in the clinical context (Figure 1). The nerve conduction velocity test for bilateral lower limbs was normal, and the repetitive nerve stimulation test was also normal.

**Figure 2:**
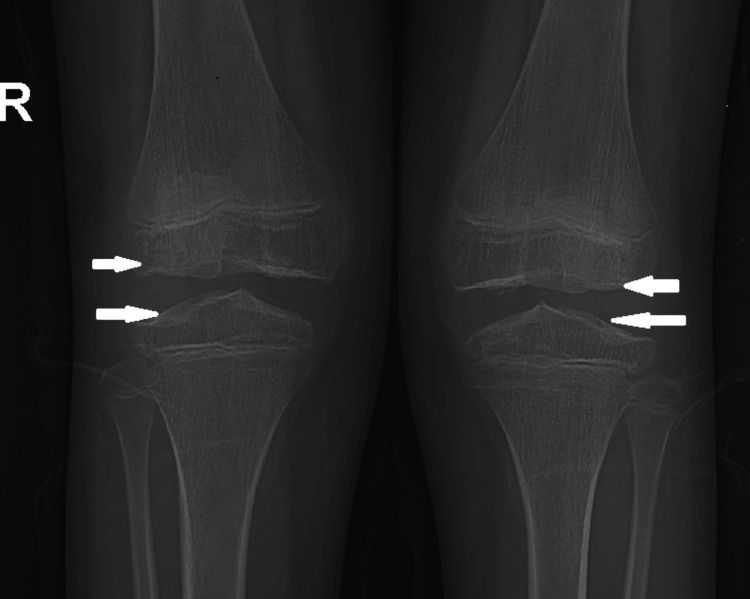
X-ray of both knee joints (AP view) showing irregularities and flattening of epiphyseal ends of femur and tibia

Due to tenderness in the right thigh muscles, an MRI of the thigh was done, which revealed a decreased muscle bulk in the right thigh. The necessity for physiotherapy was discussed with the patient, and the child was subsequently discharged. On the fourth day, she returned for a follow-up at the outpatient department. During this visit, her proximal muscle weakness had improved, and she was able to stand from a squatting position with ease. Seven days later, the child was re-admitted with acute pain in the left thigh and knee, accompanied by an inability to bear weight on the left knee. There was no reported history of injury. Mild swelling was noted in the left knee, along with tenderness in the thigh muscles. High-resolution ultrasonography of the left knee showed minimal suprapatellar bursal effusion without any effusion in the knee joint. The patient treatment started on oral diclofenac, leading to significant improvement within 72 hours, and she was discharged again. To further investigate etiology, a muscle biopsy from the left vastus lateralis was performed, and the findings were within normal limits. The whole exome sequencing was done to confirm the diagnosis, and it identified a heterozygous missense variant in exon 18 of the COMP gene. This missense mutation resulted in the substitution of tryptophan with arginine at codon 718. This mutation is pathological for multiple epiphyseal dysplasia (MED)-1. An orthopedic consultation followed to assess genotype-phenotype correlation. The orthopedic team recommended a skeletal survey, which confirmed that the epiphyseal appearance of the knee joint on X-ray aligned with the diagnosis of MED. X-ray of both knee joints revealed irregular and flattened epiphyses, indicating delayed ossification of the growth plates (Figure 2).

Genetic testing of the parents revealed a heterozygous mutation of the COMP gene in the mother, while the father's genetic test was normal. The mother reported experiencing joint pain but had no other significant clinical history. Family history and pedigree analysis indicated that no other family members were affected by this disorder. Post-test genetic counseling was done to address the family's concerns about the disease. Since this condition follows an autosomal dominant inheritance pattern, the family was advised to undergo genetic testing for their asymptomatic sibling. However, the parents declined. MED is an autosomal dominant condition but its expression may be variable. This variability was communicated to family members. The patient received counseling to avoid vigorous exercise to preserve joint function over time and was instructed about muscle-strengthening exercises. This patient is on regular follow-up with us and doing well to date with the only symptom being intermittent joint pain.

Long-term management of MED involves regular monitoring for joint degeneration and osteoarthritis. Our follow-up protocol includes orthopedic assessments, radiological monitoring of epiphyseal development, ongoing physical therapy, and pain management. Early detection of joint complications allows for timely interventions, potentially delaying the need for joint replacement surgery.

## Discussion

MED is one of the most prevalent skeletal dysplasias yet diagnosing it can be challenging. Presenting signs often include joint pain or myopathy, and the hallmark symptom of skeletal dysplasia, which is short stature, may not be evident [6]. Joint pain may lead clinicians to consider rheumatological conditions while myopathy could prompt a neurological evaluation. In our case, initial signs such as Gowers' sign and muscle tenderness led to extensive workups for myopathies. The patient's presentation was typical, characterized by fatigue during exercise (including walking and cycling) and a waddling gait. Gowers' sign was positive, and right thigh muscle tenderness was noted, with slightly elevated CPK levels. Consequently, initial differential diagnoses included myopathy and inflammatory myositis, which were ruled out through comprehensive investigations. As investigations yielded no clear answers, we proceeded with whole exome sequencing, which ultimately provided the diagnosis. The identified heterozygous mutation in the COMP gene is among the most common mutations associated with MED. This mutation is inherited in an autosomal dominant manner. Genetic testing confirmed that the mother carried the heterozygous mutation, and she had a history of very mild symptoms, such as knee joint pain. The identified COMP gene mutation demonstrates variable expressivity, as evidenced by the marked phenotypic difference between our patient and her minimally symptomatic mother. This variability has important implications for genetic counseling and highlights the need for individualized management approaches.

This case report underscores the potential for confusing presentations when distinguishing between MED and myopathy, highlighting the critical role of genetic testing in achieving an accurate diagnosis. Myopathy as a presenting symptom has been described in COMP gene autosomal dominant mutation and also documented by Markova et al. in cases of autosomal recessive mutations [3,7]. The variable penetrance associated with autosomal dominant inheritance results in phenotypical heterogeneity. Some MED cases may develop flexion deformities of the joints, which were absent in this patient, suggesting a milder phenotypic form. The bursitis observed in this case may stem from disturbed joint alignment in MED caused by ligament and tendon laxity resulting from mutations in the COMP gene, which affects the cartilage proteins in these structures. Ligament and tendon laxity can lead to decreased tone, increased injury to muscles and joints, and symptoms such as bursitis and muscle tenderness [8]. Furthermore, while Perthes disease shares similarities in that pain exacerbates with activity and radiological findings can mimic those of MED, it is primarily a unilateral condition affecting the hip joint. In contrast, MED is characterized by bilateral radiological findings involving multiple joints [9]. MED can arise from mutations in various genes and may follow either dominant or recessive inheritance patterns. Mutations in the Cartilage Oligomeric Matrix Protein (COMP) gene are linked to two common and allelic bone dysplasia: pseudo achondroplasia (PSACH) and MED-1. PSACH has a single genetic locus (19p13.11) with single gene involvement (COMP), whereas MED encompasses multiple genetic loci (19p13.11, 1p33-p33.2, 20q13.3, 5q32-q33.1, 2p24-p23, and 6q13) involving various genes (COMP, COL9A2, COL9A3, SLC26A2, MATN3, and COL9A1) [9]. Phenotypically, PSACH presents a more severe form, characterized by disproportionate short stature and significant joint laxity.

## Conclusions

MED is a generalized skeletal disorder, with the most common form of inheritance being autosomal dominant. Cardinal features of skeletal dysplasia may be absent, and initial subtle signs and symptoms can lead to misdiagnosis. This case demonstrates that MED should be considered in the differential diagnosis when evaluating children with proximal muscle weakness, even in the absence of classic skeletal dysplasia features. Early consideration of genetic testing, particularly when conventional myopathy workup is inconclusive, may prevent diagnostic delays and preserve joint function over time. Genetic testing plays a crucial role in the prompt diagnosis of such conditions.
